# Ghost rodents: Albinism in Australian rodent species

**DOI:** 10.1002/ece3.9942

**Published:** 2023-03-27

**Authors:** Darcy Watchorn, Chris Dickman, Judy Dunlop, Emmalie Sanders, Molly Watchorn, Phoebe Burns

**Affiliations:** ^1^ Centre for Integrative Ecology, School of Life and Environmental Sciences (Burwood Campus) Deakin University Geelong Victoria Australia; ^2^ Wildlife Conservation and Science Zoos Victoria Parkville Victoria 3052 Australia; ^3^ School of Life and Environmental Sciences University of Sydney Sydney New South Wales 2006 Australia; ^4^ Western Australian Feral Cat Working Group 58 Sutton St, Mandurah Mandurah Western Australia 6210 Australia; ^5^ School of Agricultural, Veterinary and Environmental Sciences Charles Sturt University Albury New South Wales 2640 Australia; ^6^ Life Sciences Zoos Victoria Parkville Victoria 3052 Australia

**Keywords:** albino, mammal, mouse, Muridae, pelage, rat

## Abstract

While almost half of all mammal species are rodents, records of albinism in free‐ranging rodents are very rare. Australia has a large and diverse assemblage of native rodent species, but there are no records of free‐ranging albino rodents in the published literature. In this study, we aim to improve our understanding of the occurrence of albinism in Australian rodent species by collating contemporary and historic records of this condition and providing an estimate of its frequency. We found 23 records of albinism (i.e., a complete loss of pigmentation), representing eight species, in free‐ranging rodents native to Australia, with the frequency of albinism being generally <0.1%. Our findings bring the total number of rodent species in which albinism has been recorded globally to 76. While native Australian species represent only 7.8% of the world's murid rodent diversity, they now account for 42.1% of murid rodent species known to exhibit albinism. We also identified multiple concurrent albino records from a small island population of rakali (*Hydromys chrysogaster*) and discuss the factors that may contribute to the relatively high frequency (2%) of the condition on this island. We suggest that the small number of native albino rodents recorded in mainland Australia over the last 100 years means that traits associated with the condition are likely deleterious within populations and are thus selected against.

## Graphical abstract

While almost half of all mammal species are rodents, records of albinism in free‐ranging rodents are very rare, and there there are no records of albinism in native Australian rodents in the published literature. In this study, we aim to improve our understanding of the occurrence of albinism in Australian rodent species by collating contemporary and historic records of this condition and providing an estimate of its frequency. We found 23 records of albinism (representing eight species) in free‐ranging rodents native to Australia. The addition of these records represents a considerable (11.8%) increase in the number of rodent species, globally, with albinism recorded, and nearly doubles the number of murid rodent species from 11 to 19 (1.3%–2.2%).

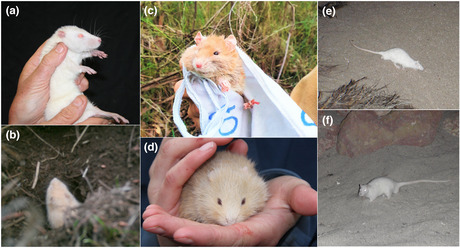

## 1 INTRODUCTION

Mammals exhibit diverse fur and skin colouration patterns that have evolved for purposes such as camouflage, intra‐ and interspecific signaling, and thermoregulation (Caro & Mallarino, 2020). Hypopigmentary congenital disorders, such as albinism and leucism, cause atypical colouration in mammals due to a deficiency of melanin—the main pigment that determines the color of skin, fur, and eyes (Hiller, 1983). While there are several forms of albinism (McCardle, 2012), true or complete albinism is characterized by the total absence of melanin, and consequently, albino mammals typically exhibit white or off‐white/straw‐colored pelage, with pale skin and pink eyes (Guillery et al., 1999; Searle, 1968). Leucism is a partial or total melanin reduction in fur or skin pigmentation (Phillips & Wilson, 1965), but unlike true albinism, it rarely affects skin and does not affect the eyes (Miller, 2005; van Grouw, 2006). Albinism is rare and typically affects one in several thousand individuals (Fernández et al., 2021). It is generally (though not always, e.g., Sage, 1962) passed on via an autosomal recessive inheritance pattern (i.e., offspring must inherit one copy of the recessive gene from both parents), and an elevated frequency of albinism typically reflects low‐genetic diversity within a population (Bensch et al., 2000; Holyoak, 1978).

Records of true albinism in free‐ranging mammals are particularly uncommon (Acevedo et al., 2009; Dunlop et al., 2019; Romero et al., 2018; Zortéa & Silva, 2018), likely due to the decreased fitness associated with the condition. For example, albinism can contribute to reduced visual ability (Guillery et al., 1999), decreased sense of smell (Keeler, 1942), increased UV sensitivity (Polanowski et al., 2012), reduced heat absorption in cold waters for marine mammals (Würsig et al., 2009), increased conspicuousness to predators (Prather, 1961), and potentially an increased likelihood of infanticide (Leroux et al., 2021). As such, free‐ranging albino mammals may be less likely to reach reproductive maturity, although there are several known exceptions (e.g. Dunlop et al., 2019; Polanowski et al., 2012; Esteve & Jefferyt, 1998).

Despite the rarity of the condition, albinism is often associated with rodents. Some Muridae species, particularly the brown rat (also known as the Norway or laboratory rat; *Rattus norvegicus*), have been selectively bred for albinism for over a century for domestication (e.g., as pets) and research purposes (Beermann et al., 2004; Castle, 1947; Castle & Allen, 1903; Hou & Protopopova, 2022; Modlinska & Pisula, 2020; Noirot, 1966; Novikova & Grigoryan, 2020). However, albinism in free‐ranging rodents appears to be uncommon and has been recorded in fewer than 2% of rodent species globally (Nations et al., 2021; Romero et al., 2018).

Australia has a diverse and ubiquitous assemblage of murid rodents that comprised at least 67 species before European invasion (Menkhorst & Knight, 2004; Roycroft et al., 2021; van Dyck & Strahan, 2008). Since that time, however, at least 13 native rodent species have become extinct, and an additional 25 species have declined enough in distribution and abundance to warrant listing as threatened at State or Federal levels (DAWE, 2021; DELWP, 2021; Northern Territory Government, 2021; OEH, 2021; South Australian Government, 2021). Four invasive rodent species also occur in the country: the brown rat, black rat (*R*. *rattus*), pacific rat (*R. exulans*), and house mouse (*Mus musculus*; Menkhorst & Knight, 2004; van Dyck & Strahan, 2008). Despite the ubiquity of rodents in Australia, to our knowledge, there are no records of native albino rodents in the published literature (Romero et al., 2018). In the gray literature, however, there is a recent mention of albinism in a population of rakali (*Hydromys chrysogaster*) on Barrow Island, Western Australia, with multiple albino rakali sightings on the island in recent years (Bettink, 2016). Barrow Island (202 km^2^) is located 90 km northeast of Onslow and represents a taxonomically distinct population that is typically smaller with lighter pelage compared to populations on the Australian mainland (Bettink, 2016).

In this study, we aim to improve our understanding of the occurrence of complete or true albinism (i.e., a total loss of pigmentation) in Australian rodent species. To achieve this, we attempt to collate all records of the condition in Australia, both contemporary and historic, using three distinct methods:

1. An examination of historic records by searching a national, digitized newspaper database, and auditing museum collections.
2. A survey of contemporary ecologists and naturalists, requesting albino rodent records and a summary of their rodent trapping data to better understand the frequency of the condition.
3. A camera trap survey of a population that is known to produce albino individuals on occasion (rakali on Barrow Island) to better understand the frequency of the condition at that location.

Notably, the patterns of albinism inheritance were not investigated as part of this study.

## 2 MATERIALS AND METHODS

### 2.1 Historic records survey

**FIGURE A1:**
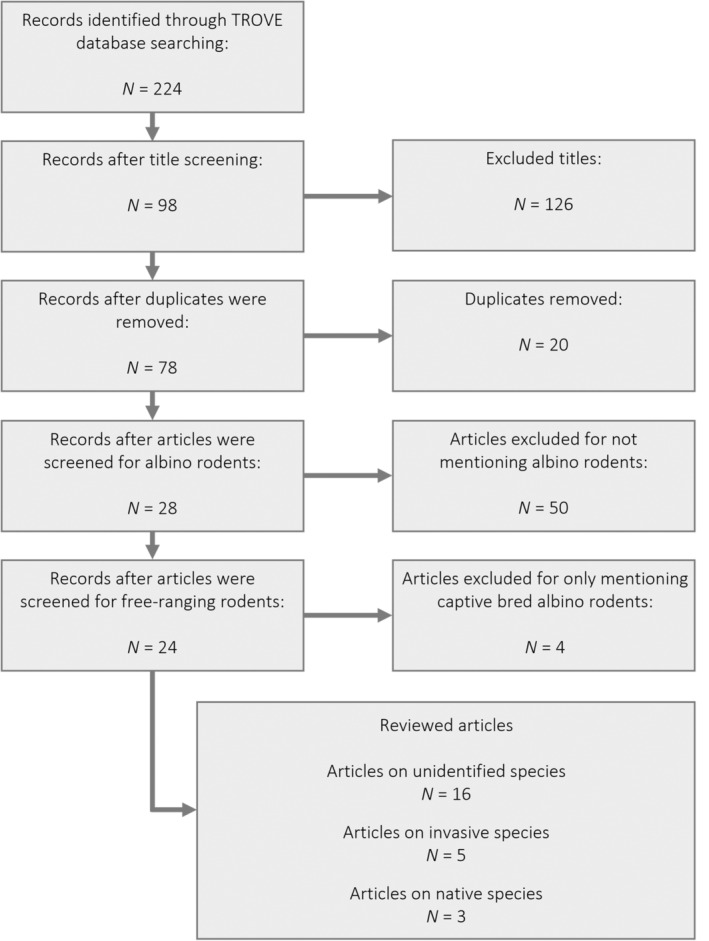
Flow diagram outlining the screening of the albino rodent literature from the Trove digitized newspaper database, resulting in the final list of 24 articles/free‐ranging albino records.

To collate historic records of albino rodents in Australia, we first completed a search of historical (gray literature) records on the Trove digitized newspaper database (https://trove.nla.gov.au/); a freely available, comprehensive digital collection of Australian newspaper articles, gazettes, and other resources dating back to 1762. We searched Trove on the May 24, 2021, using the search terms ‘albin rodent’, ‘albin rat’, and ‘albin mouse’. These search terms returned 224 articles, of which 200 (89.29%) were removed after screening as they were either duplicates or fell outside the scope of our search (e.g., records of captive rodents, records from outside Australia; Figure A1).

We then audited seven Australian State and Territory museum collections (Australian Museum, Australian National Wildlife Collection, Museum and Art Gallery of the Northern Territory, Museums Victoria, South Australian Museum, Tasmanian Museum and Art Gallery, and Western Australian Museum) for albino specimens with the assistance of mammal curators or collection managers from each institution. They searched their respective institution's collections database for any mention of the word ‘albino’ attached to native murid specimen records and visually inspected collections for any abnormally pale rodent specimens. Notably, the collection at the Queensland Museum was inaccessible for the duration of our survey; therefore, this collection was not audited.

There are several large collections of Australian rodent specimens housed in international museums. We searched international museum online databases (American Museum of Natural History [AMNH], British Natural History Museum, Field Museum of Natural History, Museum of Vertebrate Zoology [MVZ], and Smithsonian National Museum of Natural History) for records of Australian rodents with albinism but did not identify any. Some online databases lacked a notes field (AMNH, MVZ), so while Australian rodent specimens were found, there was no scope for identifying albinism if it were present. We also contacted mammalogy curators, collection managers, or research associates at several of these museums – no respondents could recall any relevant specimens within their collections, and visual inspection of the MVZ collection returned no albinos.

### 2.2 Contemporary ecologist and naturalist survey

We contacted 69 ecologists and naturalists with extensive experience conducting small mammal trapping surveys in Australia and asked whether they had any contemporary records of albino rodents. If they had recorded an albino, we asked for the year, month, and location of the detection, as well as the species, sex, and weight of the animal. To better understand the frequency with which albino individuals are detected, we also requested a summary of their rodent trapping data. Specifically, we asked for location (region and state), species, sample period (years), and sample size (i.e., the number of individuals captured during the sample period). We received responses regarding albino records from 57 ecologists and naturalists and trapping summaries from 23 ecologists and naturalists. In total, 77 species‐specific trapping summaries were provided (some ecologists provided summaries for more than one species) representing 30 species (28 native, 2 non‐native). Sample sizes for each species were combined, and we estimated the frequency of albinism only for those species with adequate sample sizes (≥1000 individuals) by dividing the number of albino individuals by the sample size. Notably, some of the larger sample sizes provided were estimated to the nearest hundred (Table A1). The complete trapping summary is available in the Appendix (Table A1). Importantly, we acknowledge that this survey of contemporary ecologists and naturalists, while extensive, was neither systematic nor exhaustive; by virtue of the scale of the task, there remain ecologists that we have not consulted and datasets we have not accessed. As such, there may be additional recent albino rodent records that are not included here.

### 2.3 Barrow Island camera survey

**FIGURE 1:**
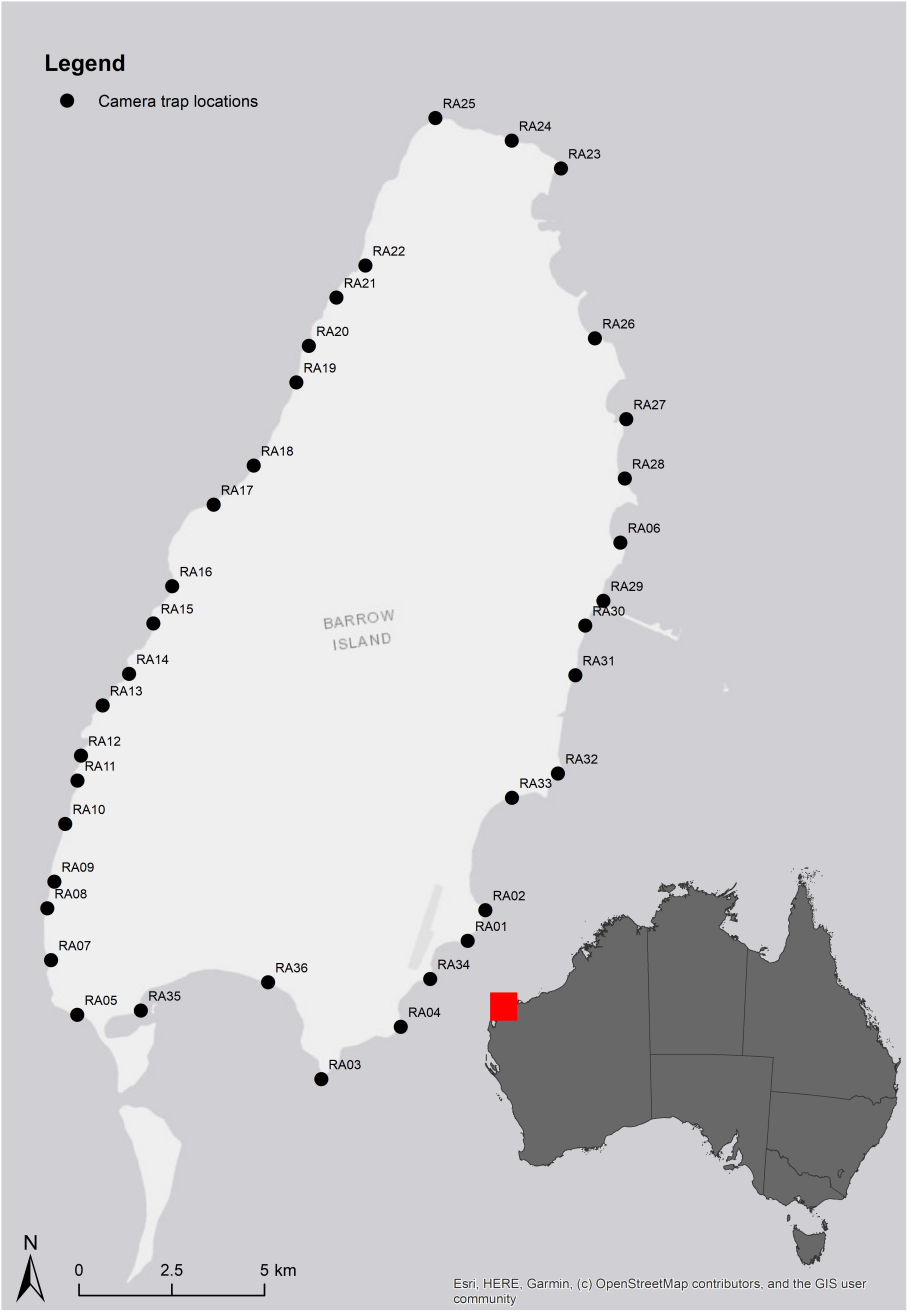
Map showing the locations of the camera traps deployed to survey rakali on Barrow Island, Western Australia.

The current population size of rakali on Barrow Island is not well‐known, but previous estimates have indicated that there may be approximately 150 individual rakali on the island (Bettink, 2016). To better understand the frequency of albinism in this population, we conducted a camera trap survey across 36 sites on the island (Figure 1). Barrow Island is a flat, dry island with hummock grassland (*Triodia* spp.) as the dominant vegetation, dotted with large termite mounds. The coastline includes rugged limestone cliffs, caves, and beaches. There is no fresh water, except during the summer monsoonal season when heavy rainfall and freshwater runoff are associated with cyclonic activity. Camera traps (*Reconyx PC900*, Reconyx, Wisconsin) were placed in limestone cliff habitat within 50 m of the shore along the islands' coast and were separated by a minimum of 750 m (mean 1377 m, 750–4700 m). Cameras were deployed in October 2019 and collected 476 days later in January 2021. During this time, cameras were operational for approximately 400 days, having SD cards and batteries changed, when possible, as Covid‐19 restricted visits to the island. Cameras sometimes failed due to heat, salt spray, cyclones, as well as “evisceration” of batteries by white‐bellied sea eagles (*Haliaeetus leucogaster*). At each site, cameras were attached to a 1.2 m picket on a 40 cm right angle bracket, facing the ground. As fishes comprise a large part of the diet of rakali, we used a perforated tin of sardines as a scent lure, secured at the base of each stake. Cameras were set to record five images per trigger event at moderate sensitivity, with no delay between possible trigger events. Each photo sequence was treated as a single point in time, and a detection event was defined as a set of images separated by at least 15 min. Images were processed and rakali were identified by *E*. *Sanders*.

## 3 RESULTS

### 3.1 Historic records survey

Our search of the Trove digitized newspaper database revealed 24 records of free‐ranging albino rodents in Australia. Of these, 16 were records of unidentified species (Table A2), five were records of invasive species, and three were records of native species (Table 1). The records of native species comprised one rakali from Victoria (recorded in 1937) and two long‐haired rats (*R. villosissimus*) from Queensland (recorded in 1940 and 1951; Table 1). Of the non‐native species, there were three records of the brown rat and two of the house mouse (Table 1).

**TABLE 1:** Records of albinism in Australian rodents sourced from the historic (gray literature and museum) survey, a contemporary ecologist/naturalist survey, and a camera trap survey for rakali on Barrow Island.

Genus	Species	Year (month)	State (region)	Sex (weight)	Survey method	Source
*Hydromys*	Rakali (*H. chrysogaster*)	1937	Vic (Melbourne)		Historic (gray literature)	M. E. Freame
		2019 (Oct)	WA (Barrow Island)		Barrow Island camera trap	Judy Dunlop, Emmalie Sanders
		2019 (Oct)	WA (Barrow Island)		Barrow Island camera trap	
		2019 (Nov)	WA (Barrow Island)		Barrow Island camera trap	
		2007	WA (Barrow Island)	F (455 g)	Contemporary	Keith Morris
		2008	WA (Barrow Island)	F (430 g)	Contemporary	
		2011	WA (Barrow Island)	M (605 g)	Contemporary	
		2011	WA (Barrow Island)		Contemporary	Pendoley Environmental
		2015	WA (Barrow Island)		Contemporary	
		2015	WA (Barrow Island)		Contemporary	
*Pseudomys*	Ash‐gray mouse (*P. albocinereus*)	1988 (Nov)	WA (Goldfields)	M (23 g)	Contemporary	Chris Dickman
	Desert mouse (*P. desertor*)	2001 (March)	QLD (Simpson Desert)		Contemporary	
	Heath mouse (*P. shortridgei*)	2012 (June)	Vic (Grampians National Park)	M	Contemporary	Susannah Hale
*Rattus*	Bush rat (*R. fuscipes*)	2021 (April)	Vic (Otway Ranges)		Contemporary	Michael Loughnan
		1980 (July)	ACT (Brindabella Ranges)	F (75 g)	Contemporary	Chris Dickman
		1991 (July)	NSW (Chichester State Forest)	M (82 g)	Contemporary	
		2021 (Jan)	Vic (Otway Ranges)	F (125 g)	Contemporary	Darcy Watchorn
	Brown rat (*R. norvegicus*)*	1891	Unknown		Historic (gray literature)	The Sydney Morning Herald
		1931	NSW (Surry Hills)		Historic (gray literature)	Daily Pictorial (Sydney)
		1945	NSW (Tallong)		Historic (gray literature)	Goulburn Evening Post
	Canefield rat (*R. sordidus*)	1939	Qld (Macknade)	M	Historic (museum)	Australian Museum
	Long‐haired rat (*R. villosissimus*)	1940	QLD (Unknown)		Historic (gray literature)	T. A. J. Carnegie & W. Wells
		1951	QLD (Unknown)		Historic (gray literature)	J. Brooke
		1991 (July)	QLD (Simpson Desert)	M (69 g)	Contemporary	Chris Dickman
		2011 (Aug)	QLD (Simpson Desert)	F (80 g)	Contemporary	
		Unknown	Unknown founder location (captive born)		Historic (museum)	South Australian Museum
		Unknown	Unknown founder location (captive born)		Historic (museum)	
*Uromys*	Giant white‐tailed rat (*U. caudimaculatus*)	pre‐1900	QLD (Unknown)		Historic (museum)	Australian Museum
*Mus*	House mouse (*Mus musculus*)*	1914	WA (North Perth)		Historic (gray literature)	The Daily News (Perth)
		1916	WA (Donnybrook)		Historic (gray literature)	The West Australian
		1999	QLD (Emerald)	F (11 g)	Contemporary	Chris Dickman

*Note*: There were 31 records of albino rodents identified from our surveys, including eight records from the gray literature survey (three native, five non‐native), four records from the museum survey (two wild‐born, two captive‐born), 16 records from the contemporary survey (15 native, one non‐native), and three records from the camera trap survey on Barrow Island. Non‐native species are denoted by an asterisk next to the species name. Month, region, and weight of specimen record are provided in parentheses where data were available. The complete contemporary survey list, which includes references and rodent species for which no albinos were recorded, can be found in the Appendix (Table A1).

Abbreviations: State abbreviations: ACT, Australian Capital Territory; NSW, New South Wales; QLD, Queensland; Tas, Tasmania; Vic, Victoria; WA, Western Australia. Sex abbreviations: M, male; F, female.

**FIGURE 2:**
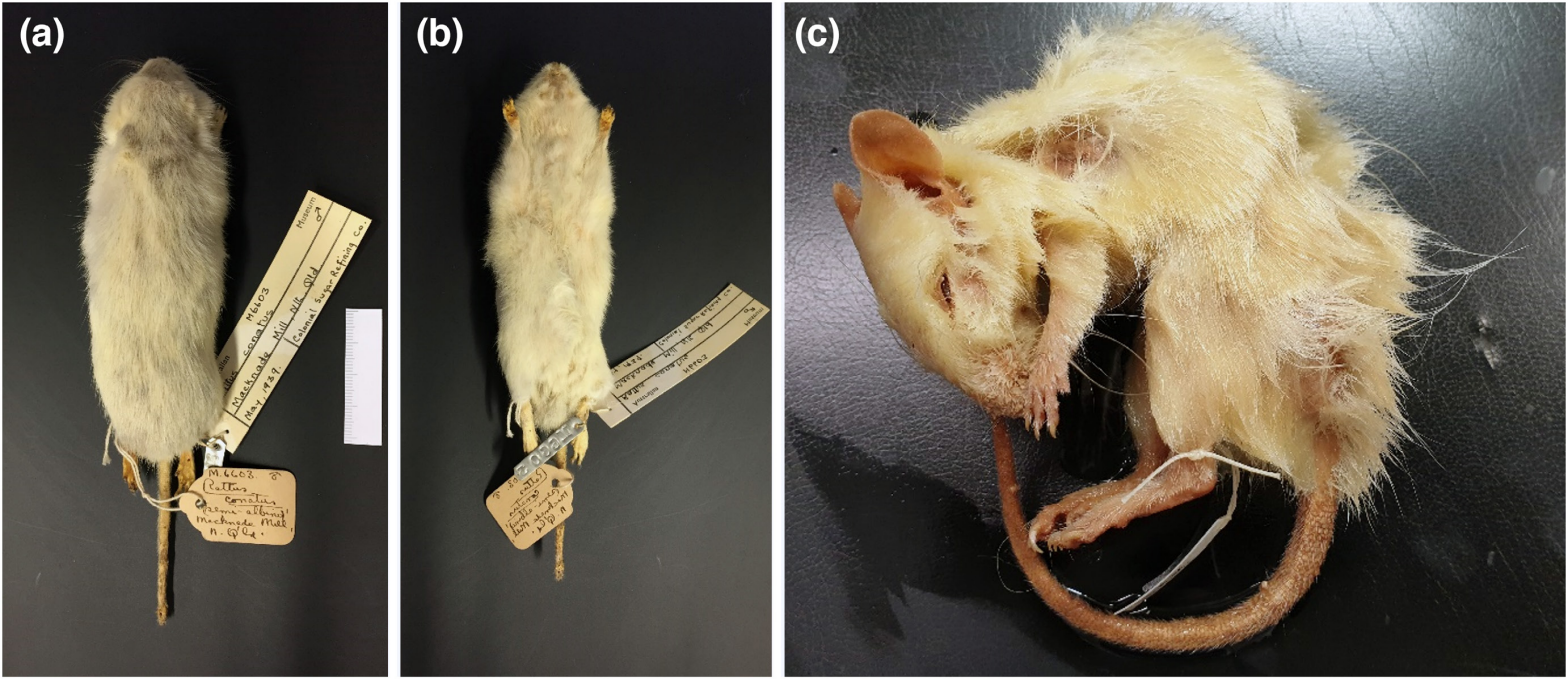
Images of two preserved albino rodent bodies identified from our survey of Australian museums: (a) dorsal image of a canefield rat (Rattus sordidus), which was labeled as ‘semi‐albino’ from the Australian Museum (New South Wales) but appears to have no pigmentation in the skin or fur except a faint pale gray at the base of the dorsal hairs (pers. comm. Sandy Ingleby; image credit Sandy Ingleby); (b) ventral image of the same canefield rat (image credit Sandy Ingleby); (c) image of a captive‐born long‐haired rat (*R*. *villosissimus*) from the South Australian Museum. No data were available on the founder source location or generation of the captive‐born animals (image credit David Stemmer).

Auditing of museum collections returned two records of wild‐caught native albino or ‘part albino’ rodent specimens (Table 1): a giant white‐tailed rat (*Uromys caudimaculatus*) and a canefield rat (*Rattus sordidus*) (Table 1) from the Australian Museum (New South Wales). The giant white‐tailed rat specimen has been lost; remaining notes label the specimen as ‘albino’ with an estimated collection date pre‐1900. The canefield rat was a skin specimen, collected from a sugar mill in 1939; it was labeled as ‘semi‐albino’, but appears to have no pigmentation in the skin or fur except a faint pale gray at the base of the dorsal hairs (pers. comm. Sandy Ingleby; Figure 2). The faint colouration and the absence of eyes (by nature of being a dry specimen) mean leucism cannot be ruled out. In addition, there were two records of captive‐born long‐haired rats from the South Australian Museum (Table 1). Of these, only one (spirit) specimen remains; it is a confirmed true albino with red eyes (Figure 2). There are, however, no data available on the founder source location or generation of the captive‐born animals.

### 3.2 Contemporary ecologist/naturalist survey

**FIGURE 3:**
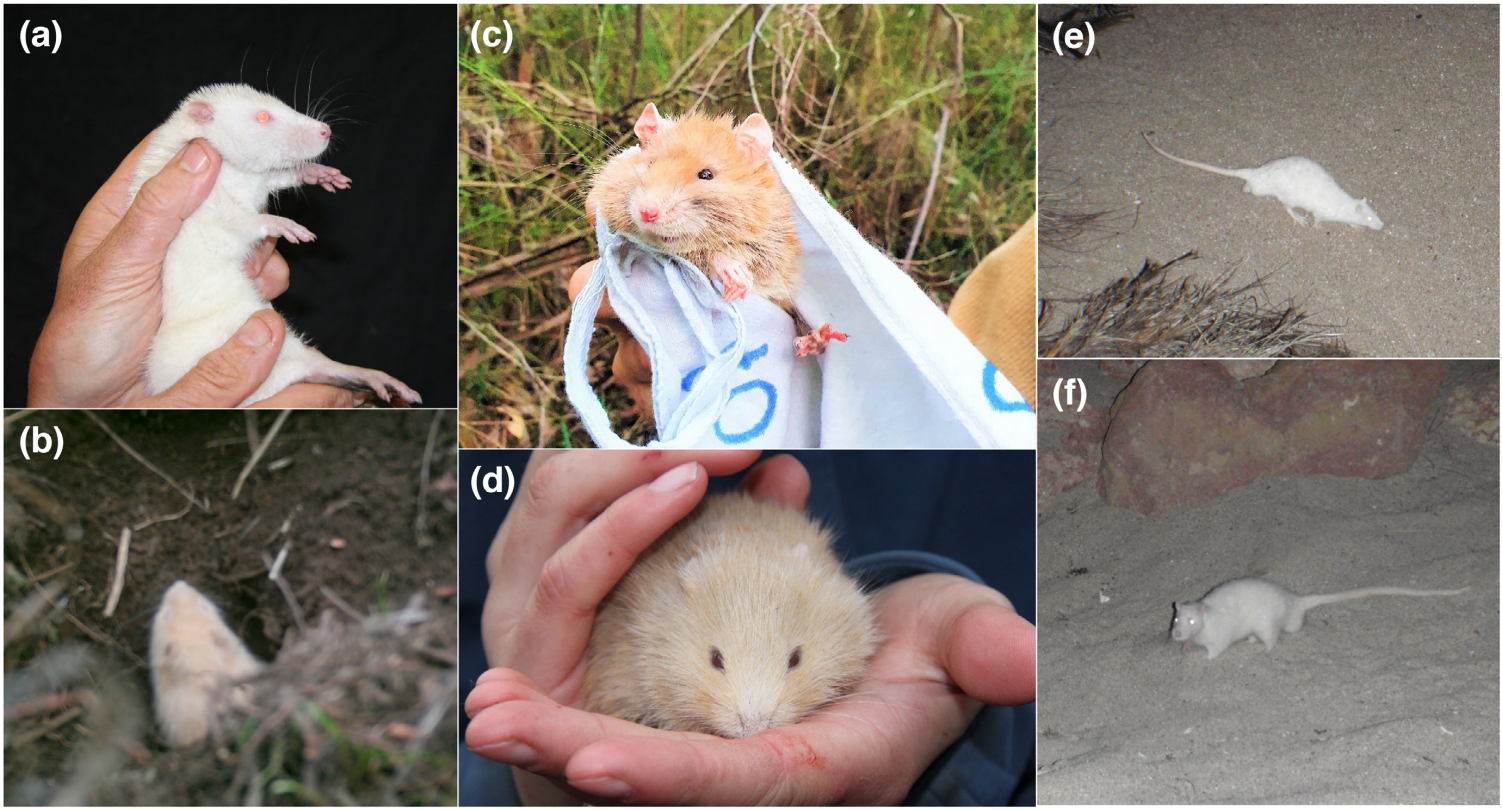
Photographs of free‐ranging albino rodents native to Australia; (a) a female albino rakali (*Hydromys chrysogaster*) on Barrow Island, Western Australia (image credit: Keith Morris); (b) an albino bush rat (Rattus fuscipes) captured on a camera trap in the Otway Ranges, Victoria (image credit: Michael Loughnan); (c) a female albino bush rat from the Otway Ranges, Victoria, exhibiting the off‐white/straw colored pelage not uncommon with albino mammals; this rat also had red, as opposed to pink, eyes (image credit: Darcy Watchorn); (d) a male albino heath mouse (*Pseudomys shortridgei*) from the Grampians National Park, Victoria (image credit: Susie Hale); (e) an albino rakali observed on Barrow Island (image credit: Pendoley Environmental); (f) an albino rakali observed on Barrow Island (image credit: Pendoley Environmental).

We collated approximately 53,159 contemporary records of live‐trapped, individual free‐ranging rodents in Australia; representing 30 species from 10 genera (Table A2). There were 16 records of free‐ranging albino rodents (dating from 1980–2021) representing six species native to Australia and one non‐native species (Table 1; Figure 3). The most common species in which albinism was recorded was the rakali with six detections (all from Barrow Island) and the bush rat (*Rattus fuscipes*) with four detections, followed by the long‐haired rat with two detections, and the heath mouse (*Pseudomys shortridgei*), ash‐gray mouse (*P. albocinereus*), desert mouse (*P. desertor*), and the non‐native house mouse each with one detection (Table 1). Most animals were live‐trapped, except for one bush rat that was recorded on a white‐flash camera trap, and three rakali that were opportunistically observed during nocturnal fauna surveys (Table 1; Figure 3). The frequency of albinism was calculated for the bush rat (0.03%), long‐haired rat (0.06%), desert mouse (0.08%), heath mouse (0.09%), and the non‐native house mouse (0.01%). The frequency of albinism for the rakali and ash‐gray mouse were not calculated, as these species had sample sizes <1000 (Table A2).

The sex ratio of albino rodents (where data were available) was equal, with six females and six males. Body weight information was available for 10 records, and the majority of individuals were relatively lightweight (Menkhorst & Knight, 2001), although no obvious signs of poor body condition or high parasite load were reported for these animals. No other physical abnormalities were reported.

### 3.3 Barrow Island camera survey

**FIGURE 4:**
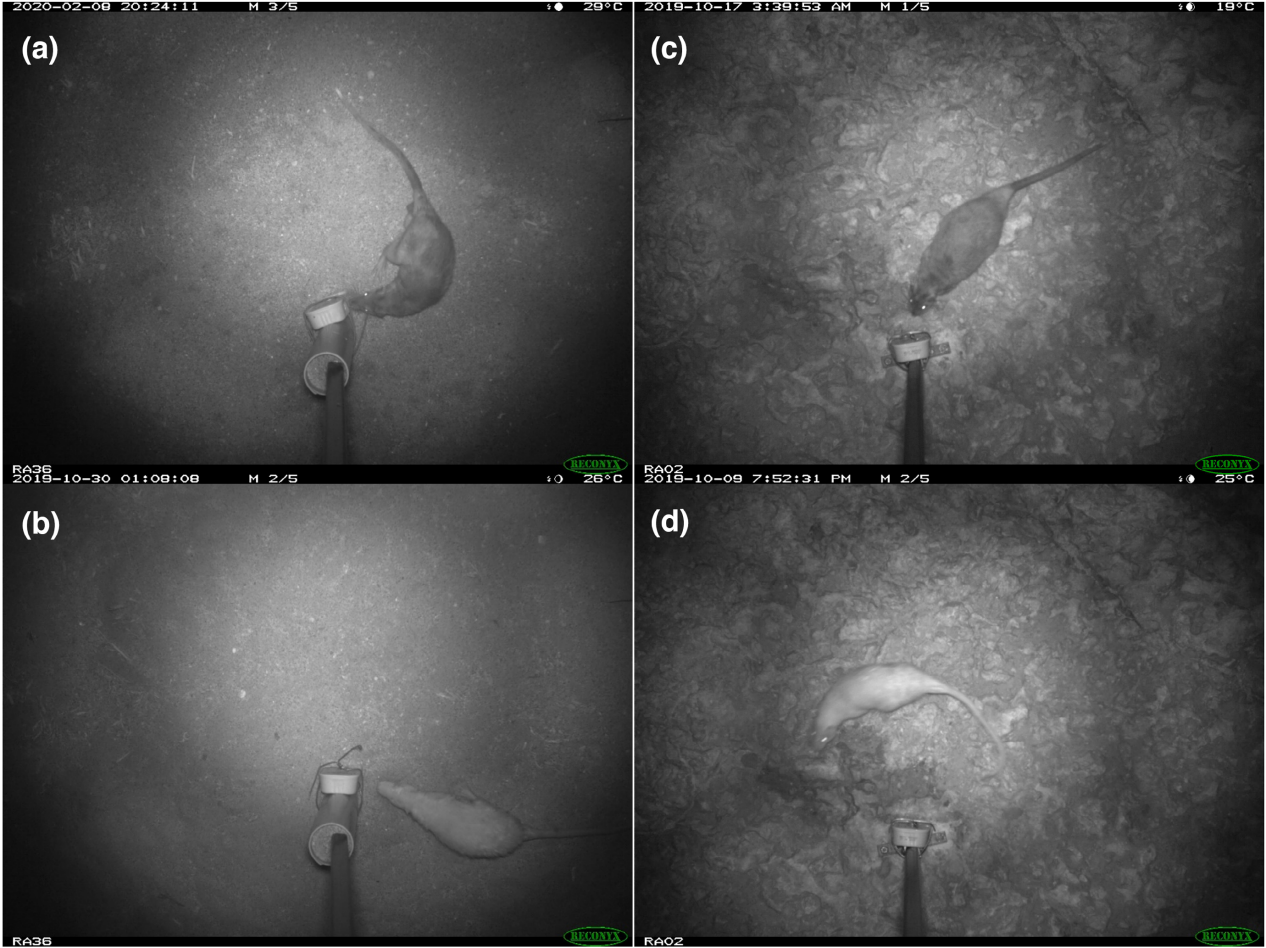
Photographs of free‐ranging rakali from the camera trap survey on Barrow Island, Western Australia, undertaken from October 2019–January 2021: (a) an image of a non‐albino rakali from site RA36, (b) an image of an albino rakali from site RA36, (c) an image of a non‐albino rakali from site RA02, and (d) an image of an albino rakali from site RA02.

We recorded a total of 285 rakali detections on Barrow Island, with rakali recorded at 34 of the 36 camera sites. There were seven detections of albino rakali (Figure 4) from a total of three sites (RA01, RA02, and RA36). Sites RA01 are RA02 were approximately 1 km apart, and while it is possible for a rakali to traverse this distance and be detected at both sites, at least two individuals could be differentiated in the camera trap images (one individual had a distinctive tail kink). Site RA36 is over 5 km from the RA01 and RA02 sites (Figure 1), and this distance is longer than overland traverses previously recorded in the species (Gardner & Serena, 1995; Harris, 1978; Vernes, 1998). Therefore, it is likely that a minimum of three albino rakali were detected on Barrow Island (Table 1), and that the frequency of albinism in this population was at least 2%, assuming a population size of 150 individuals (Bettink, 2016).

## 4 DISCUSSION

Our surveys uncovered 23 native, free‐ranging albino murid rodent records from across Australia. These records represent eight species, of which five are old endemics (rakali, giant white‐tailed rat, ash‐gray mouse, desert mouse, and heath mouse) and three are new endemics (the canefield rat, long‐haired rat, and bush rat). To our knowledge, this represents the first occasion that records of albinism in free‐ranging rodents native to Australia have been recorded in the published literature.

Records of albinism in free‐ranging rodents are rare; a review by Romero et al. (2018) found that albino specimens have been reported in just 64 of 2683 species in the order Rodentia (sensu Upham et al., 2022), with no records from Australian species. Since 2018, four more rodent species have been added to that list (Dalapicolla et al., 2020; Nations et al., 2021; Stumpp et al., 2019; van der Geer, 2019). The addition of the eight Australian species reported in this study represents a considerable (11.8%) increase in the number of rodent species, globally, with albinism recorded, and nearly doubles the number of murid rodent species from 11 to 19 (1.3% to 2.2%). We also identified seven records of albinism in two of Australia's four invasive rodent species, the house mouse and brown rat; however, albinism has been previously reported in these species (see Romero et al., 2018).

In line with Romero et al. (2018), the results from our contemporary survey also emphasize the rarity of the condition in rodents, with our 16 records (representing six native species and one non‐native species) arising from a sample of over 53,000 individuals (representing 30 species). We are assuming, however, that the encounter with an albino animal would be both obvious enough for most ecologists and naturalists to recognize and of enough interest to record. Albinism was infrequently recorded in our contemporary survey, with approximately 1 in 3300 bush rats, 1 in 1700 long‐haired rats, 1 in 1250 desert mice, 1 in 1100 heath mice, and 1 in 10,000 house mice recorded with the condition. In comparison to other estimates, however, these frequencies are relatively high. Some estimates of albinism frequency in mammals suggest 1 in every 10,000 individuals, however, species‐ and population‐specific estimates remain uncommon; most published literature on free‐ranging albino mammals reports isolated and opportunistic observations of individuals with the condition and do not estimate frequency (e.g., Acevedo et al., 2009; Dunlop et al., 2019; Ferron & Laplante, 2013; Funasaka et al., 2015; Ribeiro & de Siqueira‐Silva, 2020; Zortéa & Silva, 2018).

The frequency of albinism can, however, increase under certain conditions, such as inbreeding among small isolated populations or between closely related individuals (Caro, 2009). The rakali population on Barrow Island has been isolated for at least 8000 years and, as a result, likely has little genetic diversity and exhibits the effects of genetic drift and inbreeding depression (Bettink, 2016). Our survey of contemporary ecologists and naturalists identified six albino rakali records from Barrow Island, and our camera trap survey suggests that there were at least three albino individuals persisting on the island probably simultaneously (three records within 1 month). Our estimate that at least 1 in 50 individuals (2%) of the Barrow Island rakali population are albino is considerably higher than our other estimates from the contemporary survey, which ranged from 1 in ~1100 to 1 in 10,000 (0.01%–0.1%). In other small populations of wild mammals, low‐genetic diversity can lead to naturally occurring albino phenotypes, such as with the Japanese mole (*Mogera imaizumii*; Tsuchihashi et al., 2011), western lowland gorilla (*Gorilla gorilla gorilla*; Prado‐Martinez et al., 2013), and Asinara donkey (*Equus asinus*; Utzeri et al., 2016); however, the genetic diversity and population structure of rakali on Barrow Island are still poorly known. In contrast to the presence of dark morphs of rakali in Australia's south, albino colouration may have fewer deleterious effects for animals on Barrow Island (Bettink, 2016), which has no feral predators and is characterized by bright white sandy beaches and pale limestone coast. We note, however, that the three albino rakali detected in our camera survey were only detected in the first 4 months of the survey period (early Oct 2019 – late Jan 2020), even though the cameras remained in situ for a further 12 months (until January 2021). It is possible that these individuals suffered from predation, as the number of rakali detections was higher in the second half of the survey. As previously mentioned, Barrow Island has no introduced predators, so predation on the island would be limited to nocturnal or crepuscular birds of prey, large varanids (*Varanus giganteus*), or conspecific predation. We, along with Bettink (2016), recommend further investigation into the genetic diversity and population demographics of the rakali on Barrow Island.

Excluding the Barrow Island *Hydromys*, eight of the 14 records of albinism in Australian rodents were from arid and semi‐arid species (ash gray mouse, desert mouse, long‐haired rat) which exhibit an irruptive (boom‐bust) population cycle (Dickman et al., 1999; Greenville et al., 2012; Madsen & Shine, 1999). These dramatic fluctuations typically occur in arid and semi‐arid environments in response to pulses in primary productivity catalyzed by rainfall events, which occur unreliably in these environments (Dickman et al., 1999; Greenville et al., 2012; Madsen & Shine, 1999). Significant rainfall events can facilitate a pulse of productivity (Whitford & Duval, 2019) after which rodent populations often erupt—a period known as the ‘boom’ phase (Dickman et al., 1999, 2010; Madsen & Shine, 1999; Plomley, 1972). This intense response to rainfall is driven by high‐reproductive rates, increased survival, and the potential for females to birth multiple litters in a year. In these instances, greater resource availability and recruitment may increase both the likelihood of albino offspring being produced (higher number of births rather than an increase in proportion of albino births) and the likelihood of their survival to adulthood by improving access to food and shelter resources, hence reducing competition and predation pressures. Our contemporary records of albinism in the desert mouse and long‐haired rat were made after prolonged wet periods when populations of these species were high (Dickman et al., 2014; Greenville et al., 2013, 2016); however, an in‐depth investigation into the influence of rainfall on the frequency of albinism detections in Australian rodents was considered beyond the scope of this research.

The sex ratio reported in this study was equal; rakali, bush rats, and long‐haired rats—which account for the majority of those records—often exhibit even sex ratios (Predavec & Dickman, 1994; Press, 1987; Smart et al., 2011; Speldewinde et al., 2013; Valentine et al., 2009), although populations of long‐haired rats can skew towards either males or females (Carstairs, 1976; Predavec & Dickman, 1994). Furthermore, a survey of rakali by Bettink (2016) identified a male‐skewed sex bias on Barrow Island, albeit from a relatively small sample (*n* = 11). Most individuals with bodyweight data were relatively lightweight (Menkhorst & Knight, 2001); however, due to the small sample size of albino individuals, we were unable to infer any potential influence of albinism on sex ratio or body condition in this study, although instances of poor body condition were not reported, and we are not aware of albinism influencing the sex ratio of free‐ranging mammal populations.

## 5 CONCLUSION

Our investigation has shed light on the occurrence of albinism in Australian rodents; indeed, our addition of eight Australian species represents a considerable increase in the number of rodent species in which albinism has been recorded globally. We do note, however, that due to the rarity of albinism occurrence, we were unable to rigorously test how factors such as population dynamics, rainfall, climate, habitat type, or other possible environmental factors influence the occurrence of albinism in Australian rodents, or the fate of albino individuals. Nonetheless, we have demonstrated that albinism is an extremely rare condition in Australia and suggest that the traits associated with the condition are deleterious and thus selected against. The rakali population on Barrow Island represents a unique opportunity to further investigate whether (and if so, how) albinism confers a disadvantage on fitness and survival. The continued compilation of albinism accounts within Rodentia, both in Australia and around the world, will improve our understanding of trait‐based selection and the possible effects that this rare phenotypic condition may have on survivorship.

## AUTHOR CONTRIBUTIONS

**Darcy Watchorn:** Conceptualization (lead); data curation (lead); formal analysis (supporting); investigation (lead); methodology (lead); visualization (lead); writing – original draft (lead); writing – review and editing (lead). **Chris Dickman:** Investigation (supporting); methodology (supporting); project administration (supporting); visualization (supporting); writing – original draft (supporting); writing – review and editing (supporting). **Judy Dunlop:** Methodology (supporting); visualization (supporting); writing – original draft (supporting); writing – review and editing (supporting). **Emmalie Sanders:** Investigation (supporting); methodology (supporting); visualization (supporting); writing – original draft (supporting); writing – review and editing (supporting). **Molly Watchorn:** Investigation (supporting); methodology (supporting); visualization (supporting); writing – original draft (supporting); writing – review and editing (supporting). **Phoebe A Burns:** Investigation (supporting); methodology (supporting); project administration (supporting); visualization (supporting); writing – original draft (supporting); writing – review and editing (supporting).

## FUNDING INFORMATION

DJW was supported by a Deakin University Postgraduate Research Scholarship. No additional external funding was provided for this research.

## Data Availability

Data pertaining to historic records, contemporary ecologist/naturalist surveys, and Barrow Island camera surveys are summarized in the manuscript and are available in full in the Appendix.

## Appendix

**TABLE A1:** The total number of individual rodents captured (including albinos and non‐albinos), as identified by our contemporary ecologist and naturalist survey.

Genus	Species	Number of individuals	Ecologist/Naturalist	Reference
Conilurus	Brush‐tailed rabbit rat (*Conilurus penicillatus*)	95	Ian Radford	
Hydromys	Rakali (*Hydromys chrysogaster*)	1	Ian Radford	
Leggadina	Lakeland Downs mouse (*Leggadina lakedownensis*)	71	Ian Radford, Sarah Legge	(Legge et al., 2019)
Mesembriomys	Black‐footed tree‐rat (*Mesembriomys g*ould*ii*)	1	Ian Radford	
	Golden‐backed tree‐rat (*Mesembriomys macrurus*)	102	Ian Radford	
Mus	House mouse (*Mus musculus*)**	~9230	Billie Lazenby, Chris Dickman, Greg Hollis, Ian Radford, John Wight, Michael Driessen, Phoebe Burns, Susie Hale, Vivianna Miritis	(Lazenby et al., 2018)
*Notomys*	Mitchell's hopping mouse (*Notomys mitchellii*)	~ 125	Andrew Cockburn, Nick Clemann, Tim Doherty	(Cockburn, 1981)
	Spinifex hopping mouse (*Notomys alexis*)	~ 6000	Chris Dickman	
*Pseudomys*	Ash‐gray mouse (*Pseudomys albocinereus*)	109	Chris Dickman	
	Central pebble‐mound *Pseudomys* (*Pseudomys johnsoni*)	12	Ian Radford	
	Delicate mouse (*Pseodomys delicatulus*)	397	Ian Radford, Sarah Legge	(Legge et al., 2019)
	Desert mouse (*Pseudomys desertor*)	1331	Chris Dickman, Ian Radford	
	Heath mouse (*Pseudomys shortridgei*)	2346	Andrew Cockburn, Angela Sanders, John Wight, Julian Di Stefano, Phoebe Burns, Susie Hale	(Cockburn et al., 1981; di Stefano et al., 2011)
	New Holland mouse (*Pseudomys novaehollandiae*)	779	Billie Lazenby, Greg Hollis, Michael Driessen, Phoebe Burns	(Lazenby et al., 2018)
	Sandy inland mouse (*Pseudomys hermannsburgensis*)	~ 8121	Chris Dickman, Tim Doherty	
	Silky Mouse (*Pseudomys apodemoides*)	~1248	Andrew Cockburn, Nick Clemann	(Cockburn, 1981; di Stefano et al., 2011)
	Smoky mouse (*Pseudomys fumeus*)	399	Phoebe Burns	(Ford et al., 2003)
	Western chestnut mouse (*Pseodomys nanus*)	1045	Ian Radford, Sarah Legge	(Legge et al., 2019)
	Western mouse (*Pseudomys occidentalis*)	137	Angela Sanders	
*Rattus*	**Black rat (*Rattus rattus*)	171	John Wight, Phoebe Burns, Susie Hale	
	Bush rat (*Rattus fuscipes*)	~12,201	Angela Sanders, Barbara Wilson, Chris Dickman, Darcy Watchorn, David Lindenmayer, Chis MacGregor, Michael Loughnan, Phoebe Burns, Rosie Hohnen, Vivianna Miritis	
	Cape York rat (*Rattus leucopus*)	296	Luke Leung	(Leung, 1999b)
	Dusky rat (*Rattus colletti*)	1372	Thomas Madsen	(Madsen & Shine, 1999; Madsen, Ujvari, Shine, Buttemer, & Olsson, 2006; Madsen, Ujvari, Shine, & Olsson, 2006; Ujvari et al., 2016)
	Long‐haired rat (*Rattus villosissimus*)	3207	Chris Dickman, Keith Bellchambers (AWC)	
	Pale field rat (*Rattus tunneyi*)	1573	Ian Radford, Sarah Legge	(Legge et al., 2019)
	Swamp rat (*Rattus lutreolus*)	635	Billie Lazenby, Michael Driessen, John Wight, Phoebe Burns, Susie Hale	(Lazenby et al., 2018)
*Zyzomys*	Common rock rat (*Zyzomys argurus*)	1181	Ian Radford, Sarah Legge	(Legge et al., 2019)
	Kimberley rock rat (*Zyzomys woodwardi*)	386	Ian Radford	
*Melomys*	Cape York melomys (*Melomys capensis*)	343	Luke Leung	(Leung, 1999a)
	Grassland melomys (*Melomys burtoni*)	206	Ian Radford	

*Note*: The non‐native species are denoted by two asterisks. References are provided where applicable.

**TABLE A2:** The 24 historic records of albinism in rodents in Australia collated from the Trove digitized newspaper database that were identified to species.

Genus	Species	Year	State (region)
*Hydromys*	Rakali (*Hydromys chrysogaster*)	1937	Vic (Melbourne)
*Rattus*	Long‐haired rat (*Rattus villosissimus*)	1940	QLD (Boulia)
		1951	QLD (Connemarra)
	**Brown rat (*Rattus norvegicus*)	1891	Unknown
		1931	NSW (Surry Hills)
		1945	NSW (Tallong)
*Mus*	**House mouse (*Mus musculus*)	1914	WA (North Perth)
		1916	WA (Donnybrook)
Unknown	Unknown	1859	TAS (Bryn Estyn)
		1904	VIC (Kyneton)
		1917	NSW (Forbes)
		1917	NSW (Forbes)
		1923	VIC (Melbourne)
		1927	TAS (Myalla)
		1928	WA (Dowerin)
		1932	NSW (Unknown)
		1933	NSW (Grafton)
		1934	VIC (Lubeck)
		1936	QLD (Ipswich)
		1940	NSW (Bowral)
		1940	QLD (Mount Garnet)
		1950	NSW (Manning River)
		1951	SA (Osborne)
		1955	SA (Parkside)

*Note*: The non‐native species are denoted by two asterisks.

Abbreviations: NSW, New South Wales; QLD, Queensland; TAS, Tasmania; Vic, Victoria; WA, Western Australia.
